# The diaryl-imidazopyridazine anti-plasmodial compound, MMV652103, exhibits anti-breast cancer activity

**DOI:** 10.17179/excli2021-4323

**Published:** 2022-04-04

**Authors:** Alexis Neumann-Mufweba, Serah Kimani, Saif Feroz Khan, Kelly Chibale, Sharon Prince

**Affiliations:** 1 Department of Human Biology, Faculty of Health Sciences, University of Cape Town, Observatory, 7925, Cape Town, South Africa; 2 Department of Chemistry, Faculty of Science, University of Cape Town, Rondebosch, 7701, Cape Town, South Africa

**Keywords:** anti-plasmodial, diaryl-imidazopyridazines, drug repositioning, breast cancer, apoptosis, autophagy

## Abstract

Breast cancer is the most common malignancy in women worldwide and it remains a global health burden, in part, due to poor response and tolerance to current therapeutics. Drug repurposing, which seeks to identify new indications for existing and investigational drugs, has become an exciting strategy to address these challenges. Here we describe the anti-breast cancer activity of a diaryl-imidazopyridazine compound, MMV652103, which was previously identified for its anti-plasmodial activity. We demonstrate that MMV652103 potently inhibits the oncogenic PI4KB and PIK3C2G lipid kinases, is selectively cytotoxic to MCF7 and T47D estrogen receptor positive breast cancer cells and inhibits their ability to survive and migrate. The underlying mechanisms involved included the induction of reactive oxygen species and activation of the DNA damage and p38 MAPK stress signaling pathways. This was associated with a G1 cell cycle arrest and an increase in levels of the cyclin-dependent kinase inhibitor p21 and activation of apoptotic and autophagic cell death pathways. Lastly, MMV652103 significantly reduced the weight and metastases of MCF7 induced tumors in an *in vivo* chick embryo model and displayed a favorable safety profile. These findings position MMV652103 as a promising chemotherapeutic in the treatment of oestrogen receptor positive breast cancers.

## Introduction

Globally in 2018, there were 2,085,849 new cases of breast cancer and 626,679 breast cancer-related deaths reported, making it the leading cause of cancer related deaths among women (Bray et al., 2018[11]). Breast cancer is a heterogeneous disease which displays substantial differences in biological behaviour and therapeutic responses. It can be divided into the following four major molecular subtypes: (i) luminal A (estrogen-receptor (ER) and/or progesterone-receptor (PR) positive (+) and human epidermal growth factor receptor 2 (HER2) negative), (ii) luminal B (ER+ and/or PR+, and either HER2 positive or HER2 negative with high levels of Ki-67), (iii) triple negative/basal-like (TNBC) and (iv) HER2-enriched (Majeed et al., 2014[51]). Approximately 60-70 % of breast cancers express the estrogen-dependent ERα transcription factor which when bound by estrogens, such as 17-ß estradiol (E2), regulate cell cycle target genes including CDK2, CDK4, Cyclin D1 and the proto-oncogene c-Myc to promote cancer cell proliferation (Ciocca and Fanelli, 1997[17]; Dalvai and Bystricky, 2010[18]). The primary systemic therapy for ER+ breast cancer is endocrine therapy which is designed to counteract estrogen-induced tumor growth. For example, the gold standard tamoxifen competes with estrogen to inhibit ER activity and the aromatase inhibitors (anastrozole, exemestane, and letrozole) block the conversion of androgens to estrogen (Davies et al., 2013[20]; Pan et al., 2017[63]; Waks and Winer, 2019[84]). However, not all patients with ER+ breast tumors respond to endocrine therapy and the disease will progress or recur in a substantial number of patients who initially responded to treatment as well as those on therapy (Massarweh and Schiff, 2006[53]). It is therefore clear that ER+ breast cancers continue to pose therapeutic challenges and there is an urgent need to develop novel therapeutics to treat them (Massarweh and Schiff, 2006[53]; Gonzalez-Angulo et al., 2007[30]; Moiseenko et al., 2017[56]). 

The success of *de novo* drug development has been limited, in part, due to exorbitant research and development costs as well as the time and risks involved (Ashburn and Thor, 2004[3]; Pammolli et al., 2011[62]; Scannell et al., 2012[72]; Waring et al., 2015[85]). To overcome these challenges, drug repurposing, also broadly referred to as drug repositioning, reprofiling or rescue, which investigates existing and investigational drugs for their potential application for other diseases has gained traction. This approach has the potential to significantly shorten the drug development process as, in most cases, the drugs will have been through several stages of clinical development and will therefore have well-known pharmacokinetic and safety profiles (Ashburn and Thor, 2004[3]; Oprea and Mestres, 2012[60]; Njoroge et al., 2014[59]; Barrett and Kim, 2017[6]). To date there are a wide range of drugs that show promise for being repurposed as anti-cancer agents, with approximately 190 of them currently in late-stage clinical trials. These include acetylsalicylic acid (analgesic), metformin (treatment of type 2 diabetes), celecoxib (nonsteroidal anti-inflammatory), cholecalciferol (treatment of vitamin D deficiency), olanzapine (anti-psychotic) and zoledronic acid (treatment of bone diseases) for the treatment of, among other cancers, breast cancer (Pantziarka et al., 2018[64]). 

Using an image-based high throughput screen of a BioFocus DPI SoftFocus kinase library (SFK52) we have previously identified a novel class of imidazopyridazines with anti-plasmodial activity. Indeed, 6 of these compounds were highly potent against the K1 (multidrug resistant) and NF54 (sensitive) strains of *Plasmodium falciparum* (Le Manach et al., 2014[44]) and were found to inhibit the lipid kinases, phosphatidylinositol-4-Phosphate 3-Kinase Catalytic Subunit Type 2 Gamma (PIK3C2G) and phosphatidylinositol 4-kinase beta (PI4KB). These lipid kinases, are fundamental in regulating numerous cellular processes including cell proliferation and survival, cytoskeletal organization, membrane trafficking and glucose transport (Fruman et al., 1998[25]; McPhail and Burke, 2020[54]) and their deregulation has been associated with various cancers including colorectal (Li et al., 2015[47]), pancreatic (Harada et al., 2008[33]), ovarian (Lambros et al., 2005[40]) and breast cancers (Lapin et al., 2014[41]; Weisman et al., 2016[87]). Of interest to this study, PI4KB is highly amplified in a subset of breast cancers and contributes to their oncogenic phenotype (Waugh, 2014[86]). We therefore hypothesized that the imidazopyridazine compounds that we had identified to exhibit anti-plasmodial activity may also exhibit anti-breast cancer activity. Indeed, here we show that the diaryl-imidazopyridazine, MMV652103, exhibits potent and selective cytotoxicity against two ER+ breast cancer cell lines. Furthermore, we show that MMV652103 inhibits the ability of these breast cancer cells to survive, proliferate, and migrate and that it induces these cells to arrest in G1 and to undergo apoptosis and autophagy. We demonstrate that MMV652103 induces reactive oxygen species (ROS), triggers double-stranded DNA breaks (DSBs) and the canonical p38 MAPK stress signaling pathway. Lastly, we provide evidence that MMV652103 effectively inhibits MCF7 ER+ induced breast tumor weight and metastases in an *in vivo* chick embryo model.

## Materials and Methods

### Lipid kinase assays

A non-radiometric kinase assay (ADP-Glo™ Assay, Promega, Madison, Wi, USA) was used for measuring the kinase activity of the 2 lipid kinases, phosphatidylinositol 4-kinase beta (encoded by the PI4KB gene) and phosphatidylinositol-4-Phosphate 3-Kinase Catalytic Subunit Type 2 Gamma (encoded by the PIK3C2G gene). All kinase assays were performed in 96-well half-area microtiter plates from Greiner Bio-One (Frickenhausen, Germany) in 25 µl reaction volumes. IC_50_ values were measured by testing 10 semi-log concentrations of the compound in singlicate in each kinase assay, ranging from 1 x 10^-5^ M to 3 x 10^-10^ M. All lipid kinase assays contained 50 mM HEPES-NaOH, pH 7.5, 1 mM EGTA, 100 mM NaCl, 0.03 % CHAPS, 2 mM DTT, ATP and substrate. In addition, the assay for PI4KB contained 3 mM MnCl_2_ and the assay for PIK3C2G contained 3 mM MgCl_2_. Kinases from external vendors (Carna Biosciences Inc.; Invitrogen Corporation; Millipore Corporation) were expressed, purified and quality-controlled by virtue of the vendors readings. The reaction cocktails were incubated at 30 °C for 60 min. The reaction was stopped with 25 µl ADP-Glo reagent per well. Plates were incubated for 40 min at RT, followed by the addition of 50 µl kinase detection reagent per well and incubation for 60 min at RT. Signal was determined with a microplate luminescence reader (Victor, Perkin Elmer). All assays were performed with a Beckman Coulter Biomek 2000/SL robotic system.

For each kinase, the median value of the counts per second (cps) of three wells with complete reaction cocktails, but without kinase, was defined as "low control" (n=3). Additionally, for each kinase the median value of the cps of three other wells with the complete reaction cocktail, but without any compound, was taken as the "high control" i.e., full activity in the absence of any inhibitor (n=3). The difference between high and low control was taken as 100 % activity for each kinase. As part of the data evaluation the low control of each row of a particular plate was subtracted from the high control value as well as from their corresponding 10 "compound values". The residual activity (in %) for each well of each row of a particular plate was calculated by using the following formula:

Res. Activity (%) = 100 X [(cps of compound - low control) / (high control - low control)]

### Cell culture 

Human estrogen-receptor positive (ER+) breast adenocarcinoma cell lines, MCF7 and T47D, were maintained in Roswell Park Memorial Institute-1640 medium (Sigma Aldrich, Missouri, USA) and the human epidermal growth factor receptor 2 positive (HER2+) BT474, and triple negative (TNBC) MDA-MB-231 and MDA-MB-468 cells were maintained in Dulbecco's Modified Eagle's Medium (DMEM) (Sigma Aldrich, Missouri, USA). FG0 human skin fibroblasts (provided by Associate Professor Denver Hendricks, University of Cape Town) were maintained in DMEM while the normal breast epithelial cell line MCF12A was maintained in DMEM:nutrient mixture F-12 (DMEM/F12; 1:1; Sigma Aldrich, Missouri, USA), supplemented with 20 ng/ml epidermal growth factor, 100 ng/ml cholera toxin, 500 ng/ml hydrocortisone and 10 µg/ml insulin. All media were supplemented with 10 % fetal bovine serum, 100 U/ml penicillin and 100 µg/ml streptomycin. Cells were maintained at 37 °C in a 5 % CO_2_ and 95 % air-humidified incubator. Media was replaced every 2-3 days and cells were routinely tested for mycoplasma infection. 

### Cell treatments

Test compounds synthesized as previously described (Le Manach et al., 2014[44]) were dissolved in dimethyl sulfoxide (DMSO) (D8418; Sigma Aldrich) to give a stock concentration of 10 mM and stock solutions of the compounds were made up fresh for each treatment. Test compounds were diluted in cell culture medium to achieve the desired final concentration and a vehicle control (DMSO) of the same concentration was prepared simultaneously. Cells were treated at a confluency of 60 %. Doxorubicin (z/26/0167; Teva Pharmaceutical Industries Ltd, Israel) was used as a positive control in the ROS-Glo™ H_2_O_2_ and GSH-Glo™ Glutathione detection assays at 9.84 nM.

### Cytotoxicity assays 

Cells (3.5×10^3^-6×10^3 ^/ well) were seeded in 96-well plates and treated at a 60-70 % confluence with a range of concentrations (0-10 μM) of test compounds or vehicle for 48 h. Cell viability was measured using the 3-(4,5-dimethylthiazol-2-yl)-2,5-diphenyl-trazolium bromide (MTT) assay (11465007001; Sigma Aldrich) according to the manufacturer's instructions. Mean cell viability was calculated as a percentage of the mean vehicle control. A minimum of three independent experiments in quadruplicate were performed from which the IC_50_ was determined from sigmoidal plots using GraphPad Prism version 6.0 (GraphPad Software, California, USA). The selectivity index (SI) was determined by dividing the IC_50_ of a non-malignant cell line by the IC_50_ of a cancer cell line.

### Clonogenic survival assays

Cells were pre-cultured and treated with the indicated concentrations of MMV652103 for 24 h, washed, collected and re-plated, in drug-free medium, at 1000-2000 cells per well in 35 mm dishes, in triplicate. After 10-21 days the surviving cells were fixed with 3:1 methanol: acetic acid and stained with 0.5 % crystal violet (Sigma-Aldrich, USA) in 100 % methanol. Quantification of colonies was performed using ImageJ 1.5J8 software (USA) (Schneider et al., 2012[73]). Number of colonies was determined for each drug concentration and expressed as a percentage of the vehicle treated control. 

### Scratch motility assays

Cells were grown to 100 % confluence in 12-well tissue culture plates and the following day a sterile 2 µl pipette tip was used to make a vertical scratch in the cell monolayer of each well and the cells treated with mitomycin C (M4287; Sigma Aldrich) at a final concentration of 10 μg/ml to inhibit proliferation. Cells were subsequently treated with MMV652103 or vehicle and imaged at 0, 3, 6 and 9 h post wound formation and ImageJ v1.50i software was used to calculate the area of the scratch (Schneider et al., 2012[73]). The total areas migrated was determined by subtracting the area for a specific time point from the area measured at 0 h.

### Western blotting

Cells were exposed to MMV652103 or vehicle for 24 and 48 h. Cell lysates were prepared, and western blotting performed as previously described (Abrahams et al., 2008[1]). Primary antibodies were: PARP1/2 (sc-7150, 1:1000), p53 (sc-126, 1:1000), p21 (sc-756, 1:1000), cyclin B1 (H-433) (sc-752, 1:500) from Santa Cruz (California, USA), phospho-H2AX (#2577, 1:1000), phospho-p38 MAP Kinase (#9211, 1:1000), LC3 (#2775, 1:1000), caspase-7 (#9492, 1:1000), caspase-8 (#9746, 1:1000), caspase-9 (#9502S, 1:1000), from Cell Signaling (Boston, MA, USA), PUMA (ab9643, 1:1000) (AbCam, Cambridge, MA, USA) and p38 (M0800, 1:5000) from Sigma (St. Louis, MO, USA). Total p38 was used as a loading control because although its activity is modulated by phosphorylation, its total levels remain unaltered in response to stimuli (Raingeaud et al., 1995[68]). Densitometry readings were obtained using ImageJ v1.50i58 and protein expression levels were represented as a ratio of protein of interest/p38 loading control normalized to the vehicle treated control sample where appropriate. All blots are representative of at least three independent experiments.

### Immunofluorescence

Cells on glass coverslips were treated with either MMV652103 or vehicle for 24 and 48 h and processed for immunofluorescence with antibodies against phospho H2A.X (#2577) (1:500) or LC3B (#2775) (1:200) (Cell Signaling Technology) as previously described (Bleloch et al., 2019[8]). Cells were imaged using confocal microscopy (Carl Zeiss LSM 880 with Fast Airyscan module confocal (Oberkochen, Germany)). Multiple z layers were acquired with 1 μM step width. Images were processed using ZEN 2012 imaging software (Carl Zeiss) and maximum intensity projections were generated. For quantification, mean fluorescence was measured from at least 20 fields of view per treatment condition and pooled from three independent replicates.

### ROS-Glo™ H_2_O_2 _assay detection 

To determine whether MMV652103 induced oxidative stress in breast cancer cells, the levels of H_2_O_2_ were measured using the ROS-Glo™ H_2_O_2_ assay kit (G8820, Promega, Wisconsin, USA), according to the manufacturer's instructions. Briefly, cells (2500-4500 cells per well) were seeded in opaque 96 well plates. Cells were treated for 24 h with either vehicle or MMV652103 in a volume of 80 μl per well. After 18 h of incubation, 20 μl H_2_O_2_ Substrate Solution was added to each well. After 24 h of incubation 100 μl ROS-Glo™ Detection Solution (prepared by mixing 10 ml Luciferin Detection Reagent, 100 μl D-Cysteine and 100 μl Signal Enhancer Solution) was added to each well and incubated at RT for 20 min. After incubation, the luminescence was read using the GloMax® Explorer Multimode plate reader (Promega, Wisconsin, USA). 

### GSH-Glo^™^ Glutathione assay

The increase in ROS levels results in a decrease in the levels of antioxidants. To confirm the presence of intracellular ROS, the levels of the antioxidant, glutathione (GSH), was measured using the GSH-Glo™ Glutathione assay kit (V6911, Promega, Wisconsin, USA), according to the manufacturer's instructions. Briefly, cells (2500-4500 cells per well) were seeded in opaque 96 well plates. Cells were treated for 24 h with either vehicle or MMV652103. Thereafter, the cell culture medium was removed and 100 μl of 1X GSH-Glo™ Reagent (prepared using Luciferin-NT substrate and Glutathione S-Transferase each diluted 1:100 in GSH-Glo™ Reaction Buffer) was added to each well. The plate was then mixed briefly on a shaker and incubated for 10 min at RT. Thereafter, 100 μl reconstituted Luciferin Detection Reagent was added to each well. The plate was then incubated for 15 min RT in order to stabilize the luminescence signal. The plate was read using the GloMax® Explorer Multimode plate reader (Promega, Wisconsin, USA).

### Cell cycle analyses

Log-phase MCF7 cells were exposed to MMV652103 for 24 and 48 h and both adherent and floating cells were collected, processed as previously described and analyzed using a Becton Dickinson FACSCalibur flow cytometer (Becton Dickinson, New Jersey, USA) with a 488 nm coherent laser (Bleloch et al., 2019[8]). The data were acquired using CellQuest Pro version 5.2.1. software (Becton Dickinson) and the analyses were done using ModFit version 2.0. software (Verity Software House Inc, Maine, USA).

### Supravital staining with acridine orange (AO)

Detection of acidic vesicular organelles (AVOs) was obtained by treating cells on a glass coverslip with either MMV652103 or vehicle for 24 and 48 h. Acridine orange (Michrome No. 714; Edward Gurr Ltd, UK) was added at a final concentration of 1 μg/ml for 15 min in the dark at 37 °C. Images were obtained on a Carl Zeiss LSM 880 with Fast Airyscan module confocal (Oberkochen, Germany).

### Small interfering RNA (siRNA)

Cells were seeded at 2.5 X 10^5^ per well of a 24 well plate and transfected at 60 % confluency using with 50 or 100 nM of siLC3 (J-012846-05-0010, Dharmacon, USA) or a control (non-silencing) siRNA (1027310; Qiagen, USA)). Cells were transfected using HiPerFect® transfection reagent (Qiagen, USA) according to manufacturer's instructions.

### In vivo anti-tumor activity

All studies and procedures were conducted with prior approval of the Animal Ethics Committee of the University of Cape Town (FHS AEC REF NO:016/025) in accordance with the South African National Standard (SANS 10386:008) for the Care and Use of Animals for Scientific Purposes (SABS, 2015[71]), and guidelines from the Department of Health (2015[21]). Fertilized White Leghorn eggs were incubated at 37.5 °C with 50 % relative humidity for 9 days. At stage E9, the chorioallantoic membrane (CAM) was dropped by drilling a small hole through the eggshell into the air sac and a 1 cm^2^ window was cut in the eggshell above the CAM. Cultured MCF7 cells were detached with trypsin, washed with complete medium and suspended in PBS. An inoculum of MCF7 cells was grafted onto the CAM of each E9 egg (21 eggs were used for each condition) and at E10, tumors were detectable. MCF7 tumors were treated every 48 h (E10, E12, E14, E16) by dropping 100 μl of vehicle (0.25 % DMSO in PBS), a reference compound (Paclitaxel), or MMV652103, onto the tumour. At E18 the upper portion of the CAM was removed, transferred to PBS and the tumors cut away from normal CAM tissue and weighed. A one-way ANOVA analysis with post-tests was done. In parallel, a 1 cm^2^ portion of the lower CAM was collected to evaluate the number of metastatic cells. The relative amount of metastasis was calculated as follows: Tumor cells were grafted onto the upper chorioallantoic membrane (CAM) and during metastasis the cells invade the epithelium and basement membrane of the upper CAM and move through connective tissue into the vasculature into the lower CAM. The cells in the lower CAM are then harvested and genomic DNA is extracted and analyzed by qPCR with specific primers for Alu sequences which are only found in higher primates i.e. it allows for the distinction between human tumor cells and the host chick cells. Statistical analysis was directly performed on data from the Bio-Rad CFX Manager 3.1 software.

### Statistics

All data were obtained from at least three independent experiments (unless otherwise stated) with error bars representing standard error of the mean (SEM). Data were analyzed using GraphPad Prism version 6.0 (Graphpad Software) and a parametric unpaired t-test was performed. Significance was accepted at *p < 0.05, **p < 0.01 and ***p < 0.001.

## Results

### Cytotoxicity screening of MMV compounds

To determine if the six compounds (MMV652103, MMV665078, MMV66620, MMV674951, MMV669810 and MMV674766) identified to have anti-plasmodial activity also exhibit anti-breast cancer activity, their solubility was first tested in DMSO at a final concentration of 10 mM. As can be seen in Table 1(Tab. 1), of the 6 compounds tested, MMV652103, MMV665078, MMV66620 and MMV674951 were soluble in DMSO and were therefore selected for preliminary *in vitro* cytotoxicity studies in MCF7 breast cancer cells. Briefly, the cells were treated with a range of concentrations (2 - 20 µM) of each compound or vehicle (DMSO) for 48 h and MTT assays were performed. The half maximal inhibitory concentration (IC_50_) for each compound was calculated and MMV652103 showed the most potent cytotoxic effect with the lowest IC_50_ of 2.2 µM. To determine the selectivity index (SI) for each compound, they were also tested against non-malignant MCF12A breast epithelial cells and MMV652103 displayed the best selectivity with an SI of 3 (Table 1(Tab. 1)). Based on the promising IC_50_ and SI values obtained for MMV652103, it was chosen for further studies.

### MV652103 potently inhibits PI4KB and PIK3C2G lipid kinase activity 

**Figure 1:**
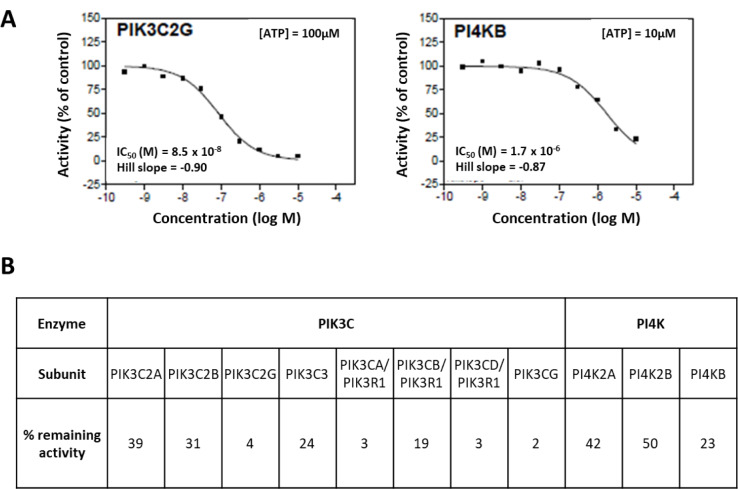
MV652103 potently inhibits PI4KB and PIK3C2G lipid kinase activity. (A) IC_50 _curves of MMV652103 from the diaryl-imidazopyridazine (SFK52) series in 2 lipid kinase assays, PIK3C2G and PI4KB. (B) Table showing the percentage remaining activity of PI4KB and PIK3C2G, and their respective subunits, after treatment with MMV652103 from the diaryl-imidazopyridazine (SFK52) series at a concentration of 10 µM. The IC_50_ values were measured by testing 10 semi-log concentrations of the compound in singlicate in each kinase assay, ranging from 1 x 10^-5^ M to 3 x 10^-10^ M.

To determine which kinases are targeted by our 6 frontrunner compounds a non-radiometric assay (ADP-Glo™ Assay, Promega, Madison, Wi, USA) was used to measure their effect on the activity of the two human lipid kinases, phosphatidylinositol 4-kinase beta (PI4KB) and phosphatidylinositol-4-phosphate 3-kinase C2 domain-containing gamma polypeptide (PIK3C2G). Based on the results from our cytotoxicity assays (Table 1(Tab. 1)) only the data for MMV652103 are shown (Figure 1(Fig. 1)). MMV652103 was found to yield an IC_50_ of 0.085 µM for PIK3C2G and 1.7 µM for PI4KB (Figure 1A(Fig. 1)) and after 10 µM MMV652103 treatment, only 23 % PI4KB activity and a mere 4 % PIK3C2G activity was detected (Figure 1B(Fig. 1)). In addition, 10 µM MMV652103 treatment resulted in 42 % PI4K2A and 50 % PI4K2B subunit activity and the activity for the 7 PIK3C2G subunits tested ranged from 39 % to 2 %. These data clearly indicate that MMV652103 potently targets the oncogenic lipid kinases PI4KB and PIK3C2G.

### MMV652103 exerts potent and selective cytotoxicity in ER+ breast cancer cells

**Figure 2:**
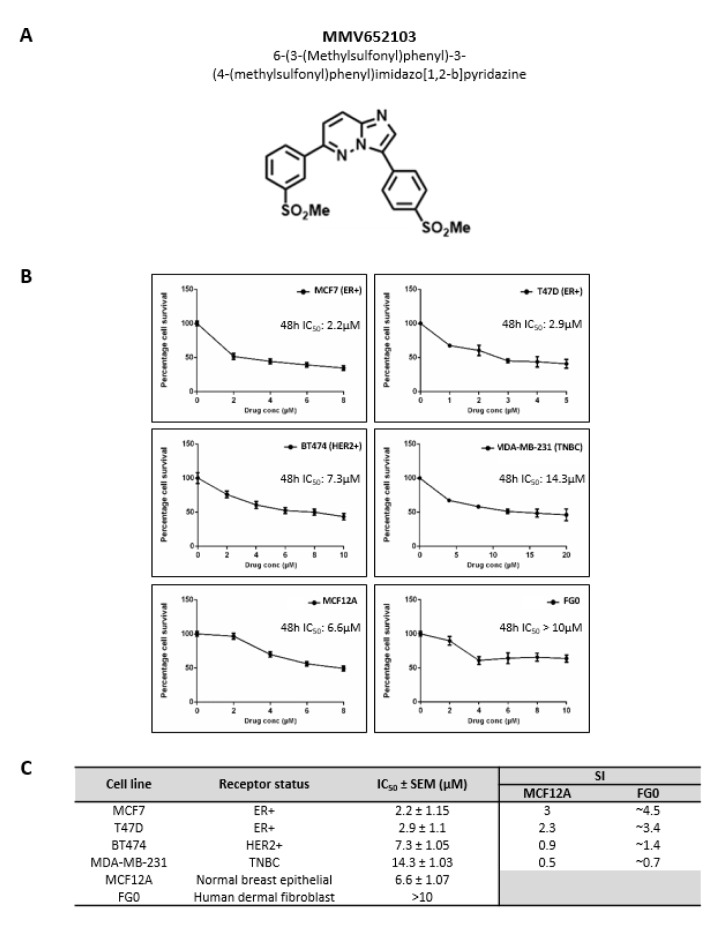
MMV652103 exerts selective cytotoxicity in estrogen receptor positive (ER+) breast cancer cells. (A) Chemical name and structure of MMV652103. (B) MTT cell viability assays of MCF7 and T47D (ER+), BT474 (HER2+) and MDA-MB-231 (TNBC) breast cancer cell lines, MCF12A (normal breast epithelial cell line) and FG0 (human skin fibroblast cells), treated with a range of MMV652103 concentrations (1-20 µM) or vehicle (DMSO) for 48 h. Graphs show mean cell viability as a percentage of vehicle control ± SEM for each concentration of MMV652103 determined from three independent experiments performed in quadruplicate. (C) Tabled summary of the IC_50_ concentrations of MMV652103 obtained for each cell line ± SEM, together with selectivity indices (SIs) which were determined for each breast cancer cell line by dividing the IC_50_ of each non-malignant cell line by the IC_50_ of the respective breast cancer cell line.

When MMV652103 (Figure 2A(Fig. 2)) was tested in a range of breast cancer cells with varying hormone receptor status the following IC_50_ values were obtained: 2.2 µM for MCF7 (ER+), 2.9 µM for T47D (ER+), 7.3 µM for BT474 (HER2+), 14.3 µM for MDA-MB-231 (TNBC) (Figure 2B(Fig. 2)). Interestingly, the SI values calculated using the MCF12A cells show that MMV652103 was only selective against the ER+ MCF7 and T47D cell lines (SI values of ≥2) and therefore it was further characterized in these cells (Figure 2C(Fig. 2)). Although an exact IC_50_ for MMV652103 was not reached for the FG0 cells, the predicted IC_50_ was >10 μM and therefore the SI values for MCF7 and T47D are also likely to be >2 (Figure 2B(Fig. 2)).

### MMV652103 inhibits the ability of MCF7 and T47D ER+ breast cancer cells to survive and migrate

**Figure 3:**
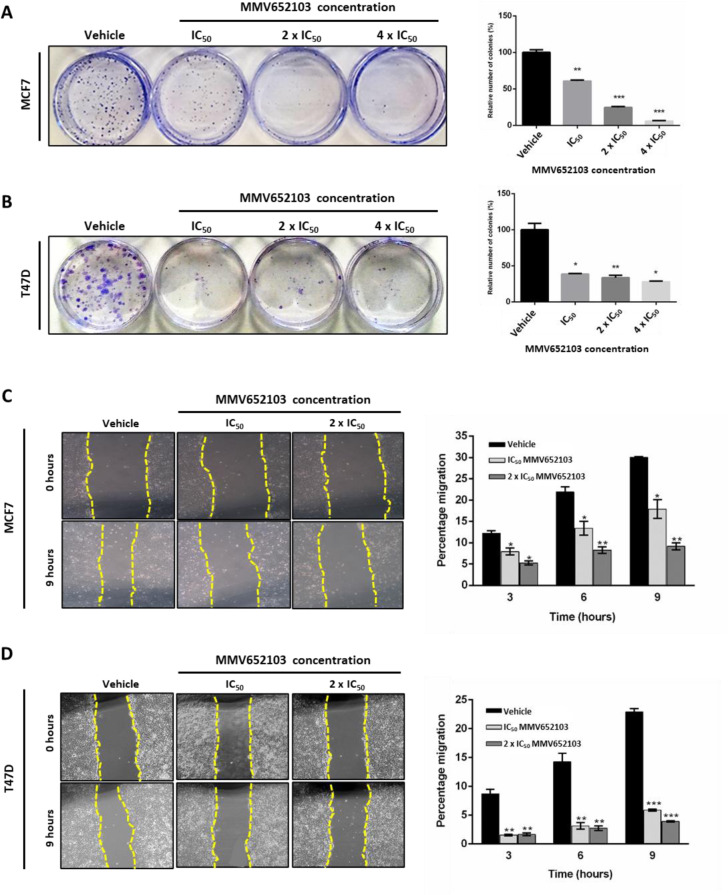
MMV652103 inhibits long-term survival and migration of MCF7 and T47D ER+ breast cancer cells *in vitro*. Representative images and quantification of clonogenic assays of (A) MCF7 and (B) T47D cells which were treated with vehicle (DMSO), IC_50_, 2 x IC_50_ and 4 x IC_50_ MMV652103 for 24 h. Colonies were stained with crystal violet and images from three independent experiments were quantified using ImageJ. The graphs represent the relative number of colonies in percentage (relative to the vehicle control). Quantification and representative images (×200; EVOS XL AMEX1000 Core Imaging System) of scratch motility assays of (C) MCF7 and (D) T47D cells treated with IC_50_ and 2 x IC_50 _concentrations of MMV652103 or vehicle for 9 h. Cells were imaged at 0, 3, 6, and 9 h post wound formation. Total area migrated was calculated by subtracting the wound area at each time point from the wound area at time 0 h, which is represented in the graphs as mean area migrated ± SEM pooled from three independent experiments. Data were analyzed using GraphPad Prism 6.0 and a parametric unpaired t-test was performed where *p < 0.05, **p < 0.01, ***p < 0.001.

Metastatic breast cancer is often associated with treatment resistance (Gonzalez-Angulo et al., 2007[30]; Redig and McAllister, 2013[69]) and therefore we determined if MMV652103 was able to inhibit the long-term survival and migration of the MCF7 and T47D breast cancer cells using the clonogenic and scratch motility assays respectively. The results show that MMV652103 treatment led to a dose-dependent reduction in the number of MCF7 and T47D cell colonies (Figure 3A, B(Fig. 3)) and a significant reduction in MCF7 and T47D cell migration at all concentrations and timepoints tested (Figure 3C, D(Fig. 3)). Taken together, these data demonstrate that MMV652013 inhibits the ability of MCF7 and T47D ER+ breast cancer cells to survive and migrate.

### MMV652103 induces ROS production in breast cancer cells

**Figure 4:**
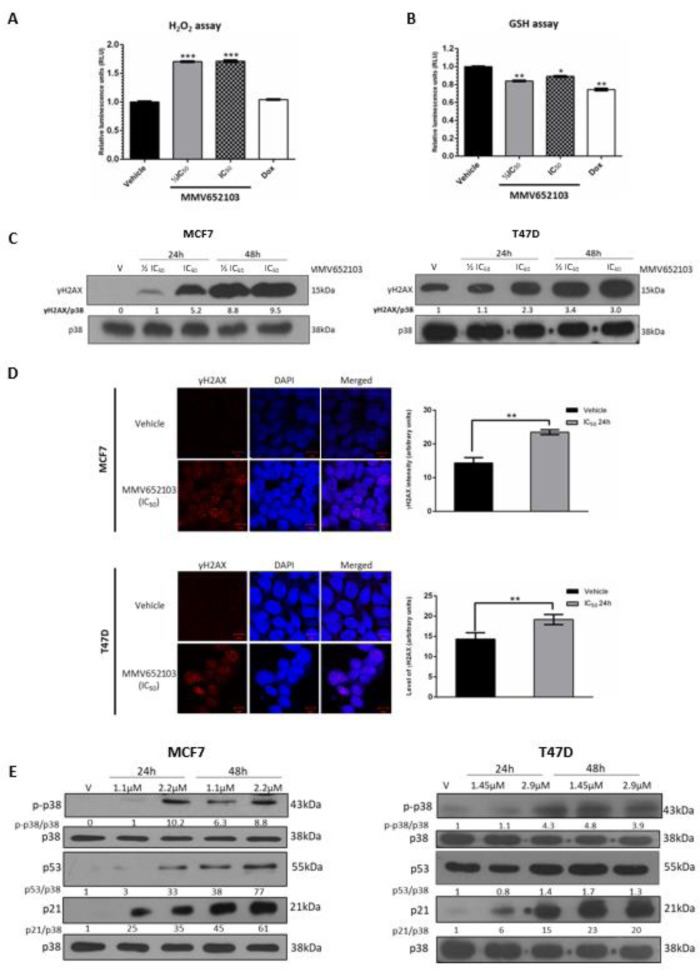
MMV652103 induces the production of reactive oxygen species (ROS) and induces double-strand DNA breaks. Levels of cellular (A) H_2_O_2 _and (B) GSH in MCF7 cells after 24 h treatment with ½ IC_50_ and IC_50_ concentrations of MMV652103. Doxorubicin at its IC_50_ concentration (9.84 nM) for MCF7 cells was used as a control. Graphs show the mean H_2_O_2_ and GSH levels of three independent experiments ± SEM. (C) MCF7 and T47D cells were treated with vehicle (DMSO), ½ IC_50_ and IC_50_ for 24 and 48 h and western blotting performed to determine the levels of γH2AX. (D) Immunocytochemistry of MCF7 and T47D cells treated with vehicle (DMSO) and IC_50_ for 24h. Images were captured under a confocal microscope (Zeiss, Germany) at 600X magnification and are representative of five randomly selected fields of view for each treatment (scale bars indicate 10 μM). The results were analyzed and show the average intensity (arbitrary units) for each treatment. (E) Western blot analyses of protein harvested from cells treated as indicated and incubated with antibodies against p-p38, p53 and p21. For all western blots total p38 was used as a loading control. Densitometry readings were obtained using ImageJ and protein expression levels are represented as a ratio of protein of interest/p38 normalized to the vehicle control sample. Blots are representative of at least three independent experiments. Data were analyzed using GraphPad Prism 6.0 and a parametric unpaired t-test was performed where *p < 0.05, **p < 0.01, ***p < 0.001.

ROS are chemically reactive oxygen radicals or non-radical oxygen derivatives, and excessive amounts of ROS can cause oxidative damage to lipids, proteins, and DNA. Therefore, anti-cancer agents that increase intracellular ROS are desirable because they can induce cancer cells to arrest in the cell cycle, undergo senescence and apoptosis (Lau et al., 2008[42]; Trachootham et al., 2009[78]; Teppo et al., 2017[77]). We therefore determined the impact of MMV652103 on ROS production by measuring the levels of hydrogen peroxide (H_2_O_2_) in MMV652103 treated MCF7 cells. H_2_O_2 _is convenient to assay as it has the longest half-life of all ROS in cultured cells and various ROS are often converted to H_2_O_2_ within cells (Rudzka et al., 2015[70]; Turrens, 2003[80]; Newsholme et al., 2012[58]). Doxorubicin, one of the most widely used chemotherapeutic drugs, was included in this assay because the generation of ROS is one of the main mechanisms by which it exerts cytotoxic effects in cancer cells, including breast cancer cells (Mizutani et al., 2005[55]; Pilco-Ferreto and Calaf, 2016[66]). The results show that, at both concentrations of MMV652103 tested, there was a significant increase in the levels of H_2_O_2_ which was higher than that obtained for the reported IC_50_ of doxorubicin (9.84 nM) (Figure 4A(Fig. 4)) (Yang et al., 2013[89]). To confirm these results, we measured the effect of MMV652103 on levels of glutathione (GSH) which plays an important role in protecting cells from the damaging effects of ROS. In the presence of ROS, GSH levels drop due to either oxidation or reaction with the thiol group in its cysteine moiety and therefore the levels of GSH are a useful indicator of oxidative stress (Venè et al., 2011[82]; Jin et al., 2016[35]). A significant decrease in the levels of GSH was observed in MCF7 cells treated with IC_50 _doxorubicin and both concentrations of MMV652103 tested (Figure 4B(Fig. 4)). These results suggest that MMV652103 may inhibit breast cancer cell viability by inducing an increase in ROS production and reducing levels of GSH.

### MMV652103 activates the DNA damage and p38 MAPK pathways in MCF7 and T47D ER+ breast cancer cells

To determine whether the MMV652103-induced ROS production led to DNA double-stranded breaks (DSBs), MCF7 and T47D cells treated with MMV652103 were subjected to western blotting and immunocytochemistry with antibodies to γH2AX, a robust marker of DSBs. The results revealed a dose- and time-dependent increase in levels of γH2AX (Figure 4C(Fig. 4)) as well as accumulation of distinct γH2AX foci in the nuclei of MMV652103 treated breast cancer cells (Figure 4D(Fig. 4)). Interestingly, in the western blots shown in Figure 4C(Fig. 4), relative to vehicle treated MCF7 cells, which had almost undetectable levels of γH2AX, vehicle treated T47D cells expressed high levels of γH2AX. It is worth noting that while MCF7 cells express wild type p53, T47D cells express mutant non-functional p53 and cells with mutant p53 have been reported to have higher basal expression of γH2AX (Yu et al., 2006[90]). Notably, ROS is capable of directly mediating the phosphorylation and activation of the p38 MAPK stress signaling pathway which converge on p53 and p21 to execute tumor suppressor functions in a broad spectrum of cancers (Avisetti et al., 2014[4]; Choi et al., 2018[15]; Kim et al., 2018[38]). Our results show that MMV652103 also increased the levels of phosphorylated p38 which suggests that MMV652103 treatment induced DNA damage and activated the p38 MAPK stress pathway (Figure 4E(Fig. 4)). Both these signaling pathways converge on p53, a well-known transcriptional activator of the cyclin dependant kinase inhibitor p21 which is key to inducing cell cycle arrests and/or apoptosis under conditions of stress (Sionov and Haupt, 1999[76]; Shi et al., 2012[74]; Deryabin et al., 2016[22]). While MCF7 cells treated with MMV652103 displayed a dose- and time-dependent increase in the levels of p53, MMV652103 had no effect on levels of p53 in T47D cells which express mutant non-functional p53 (Figure 4E(Fig. 4)) (Vojtĕsek and Lane, 1993[83]). MMV652103 treatment, however, increased p21 levels in both cell lines which suggests that its effects on p21 may be p53-dependent and -independent. It is worth noting that activated p38 can also directly increase levels of p21 which suggests that this may be the p53-independent mechanism involved (Gartel and Tyner 2002[27]). While up to 80 % of human cancers harbor *p53* mutations, only 20 % of all breast cancers have mutations in *p53* and they are very rare in ER+ breast cancers (Berger et al., 2013[7]; Ungerleider et al., 2018[81]). The rest of the experiments was therefore performed in the MCF7 cells.

### MMV652103 triggers a G1 cell cycle arrest in MCF7 breast cancer cells

**Figure 5:**
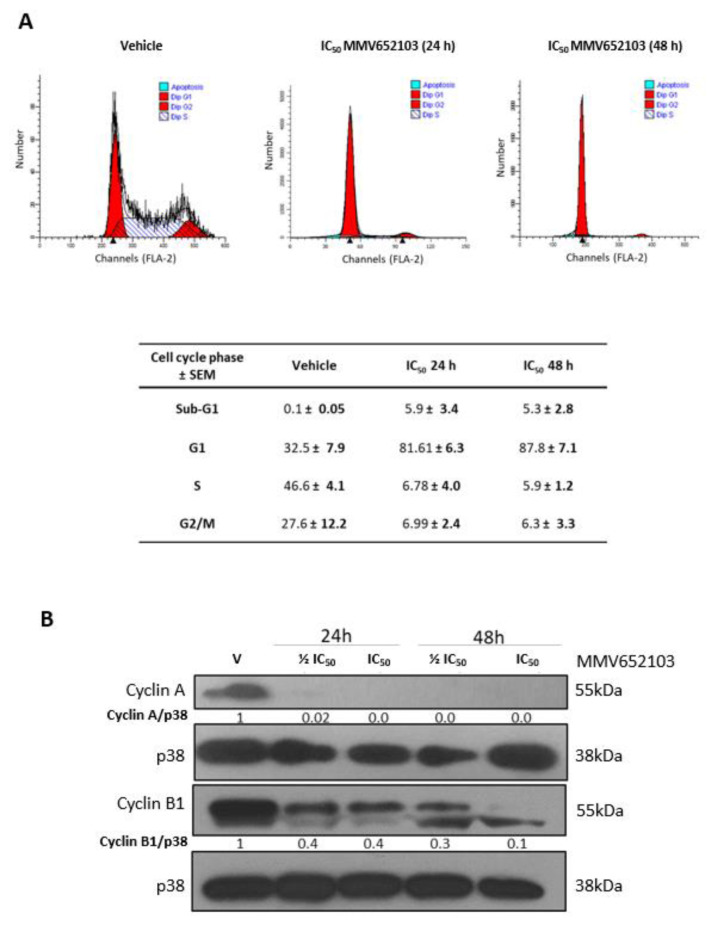
MMV652103 induces a sub-G1 peak and G1 cell cycle arrest in MCF7 breast cancer cells. (A) Representative cell cycle profiles of MCF7 cells exposed to vehicle or IC_50 _MMV652103 for 24 and 48 h as determined by staining cells with propidium iodide and measuring their DNA content by flow cytometry. The table shows the proportion of cells at each phase of the cell cycle expressed as a percentage of the total number of cells analyzed ± SEM pooled from three independent experiments. (B) Western blot analyses of protein harvested from cells treated as indicated and incubated with antibodies against cell cycle markers cyclin A and cyclin B1 and p38 was used as a loading control. Densitometry readings were obtained using ImageJ and protein expression levels are represented as a ratio of protein of interest/p38 normalized to the vehicle control sample. Blots are representative of at least three independent experiments.

To investigate the effect of MMV652103 on MCF7 cell cycle progression we performed flow cytometry and western blotting with antibodies to cyclin A and cyclin B1 which are needed for transition through the S and G2/M phases respectively (Pagano et al., 1992[61]; Gavet and Pines, 2010[28]). We show that MMV652103 induced sub-G1 peaks and arrested cells in G1 (Figure 5A(Fig. 5)) and reduced cyclin A and cyclin B1 levels (Figure 5B(Fig. 5)). The sub‐G1 or hypodiploid peak suggests that MMV652103 induces cell death because fragmentation of internucleosomal DNA is a key feature of apoptosis (Darzynkiewicz et al., 1997[19]; Kajstura et al., 2007[37]). 

### MMV652103 triggers intrinsic and extrinsic apoptosis in MCF7 breast cancer cells

**Figure 6:**
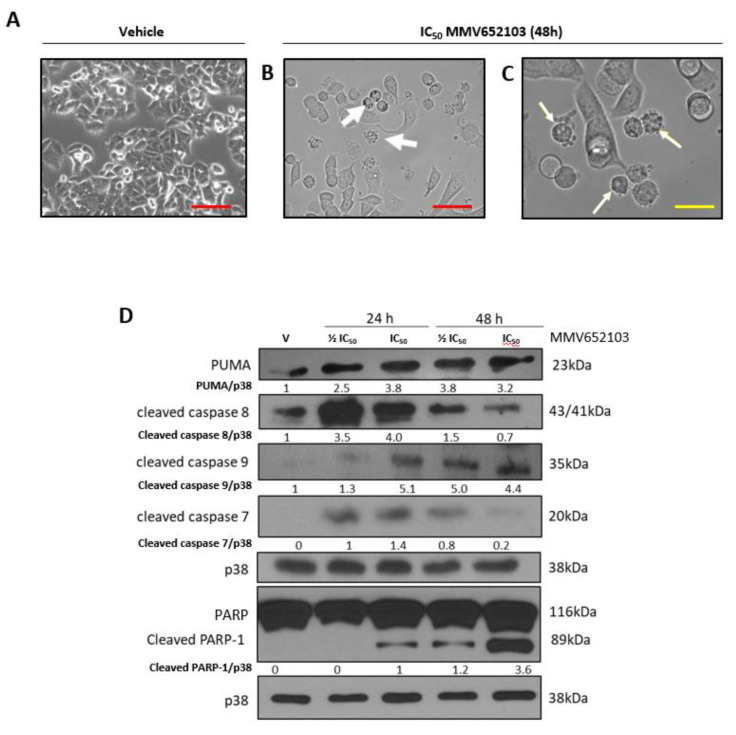
MMV652103 induces apoptosis. Representative phase-contrast photomicrographs are shown of MCF7 cells treated with (A) vehicle (DMSO) and (B and C) IC_50_ MMV652103 for 48 h (EVOS XL AMEX1000 Core Imaging System; red scale bar = 100 µm (100X), yellow scale bar = 25 µm (200X)). White arrows indicate morphological features of apoptosis including cell shrinkage, membrane blebbing and pyknosis. (D) Western blotting of protein from MCF7 cells treated with ½ IC_50_ and IC_50 _concentrations MMV652103 and antibodies to key apoptotic markers as indicated. Total p38 was used as a loading control. Densitometry readings were obtained using ImageJ and protein expression levels are represented as a ratio of protein of interest/p38 normalized to the vehicle control sample. Blots are representative of at least three independent experiments.

Light microscopy confirmed that MMV652103 induced characteristic features of apoptosis including membrane blebbing and cell shrinkage (Figure 6A-C(Fig. 6)). Furthermore, MMV652103 treatment led to increased levels of the pro-apoptotic factors, PUMA, cleaved caspase-8 and caspase-9 as well as levels of cleaved caspase-7 and its substrate poly (ADP-ribose) polymerase (PARP) (Figure 6D(Fig. 6)). While levels of cleaved caspase-8, the effector of the extrinsic apoptotic pathway, were at their highest after 24 h of MMV652103 treatment, levels of cleaved caspase-9, the effector of the intrinsic apoptotic pathway, appeared to be highest after 48 h of treatment. These results are important since many cancers harbor mutationally inactive *p53*, and consequently a compromised intrinsic pathway, which reduces their sensitivity to conventional treatment regimens. Any drug that therefore induces both apoptotic pathways may prove to be more effective. 

### MMV652103 induces autophagic cell death in MCF7 breast cancer cells

**Figure 7:**
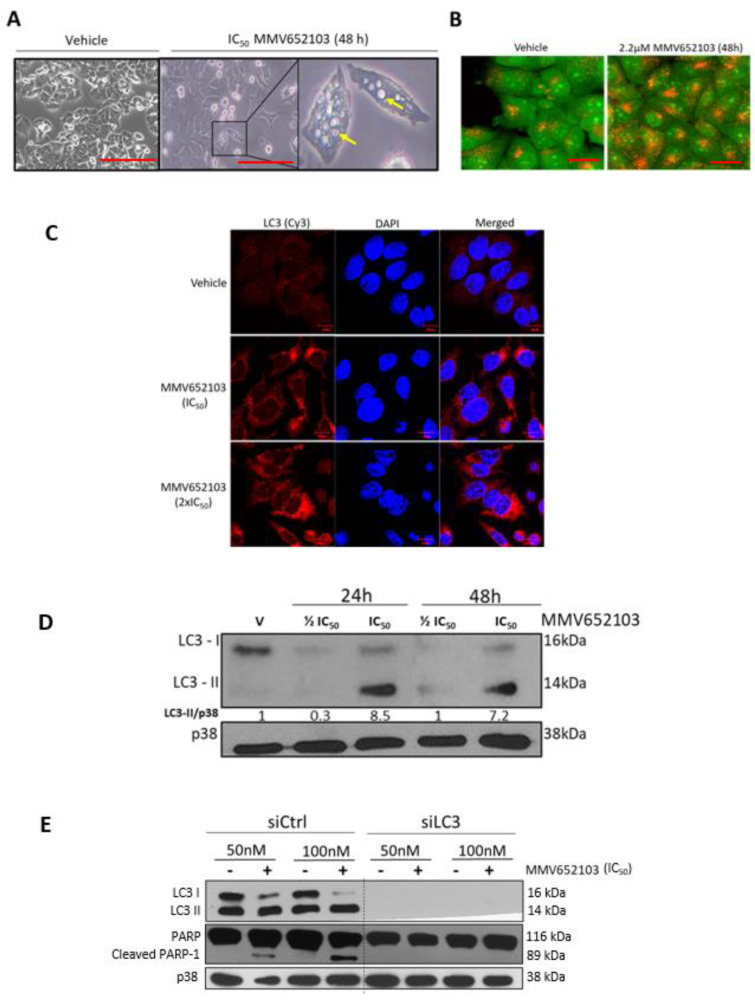
MMV652103 induces autophagy in MCF7 breast cancer cells. (A) Representative phase-contrast photomicrographs of MCF7 cells treated with vehicle (DMSO) and IC_50_ MMV652103 for 48 h. Yellow arrows indicate large vacuolar structures reminiscent of autophagic vesicles. (B) Representative confocal images (100X; EVOS XL AMEX1000 Core Imaging System; red scale bar = 100 µm) of MCF7 cells treated for 48 h with MMV652103 and stained with acridine orange (AO) which depicts the presence of acid vesicular organelles (AVOs, yellow to orange puncta). (C) Representative maximum intensity projection confocal immunofluorescence images (630X; Zeiss LSM 510; scale bar is 10 μm) from three independent experiments of MCF7 treated with IC_50_ and 2 x IC_50 _MMV652103 or vehicle for 48 h and incubated with LC3 primary antibody, fluorophore conjugated Cy3 secondary antibody and nuclei were stained with DAPI. (D) Western blotting of proteins from MCF7 cells treated with MMV652103 at IC_50_ and 2 x IC_50_ concentrations with LC3ß cleavage (LC3-I and LC3-II) analyzed at the indicated timepoints. (E) Cells transfected with non-silencing siRNA (sictrl) or LC3 specific siRNA (siLC3) and treated with MMV652103 at the IC_50_ concentration for 24 h and western blot analysis performed for the indicated proteins. For all western blot analysis total p38 was used as a loading control and densitometry readings were obtained using ImageJ. Protein expression levels are represented as a ratio of protein of interest/loading control normalized to a control sample. Blots are representative of at least three independent experiments.

We next sought to determine whether MMV652103 induces autophagy since autophagic-like vacuolar structures and acidic vesicular organelles (AVOs) were observed in MCF7 cells treated with the compound (Figure 7A, B(Fig. 7)). Results from immunocytochemistry show that MMV652103 treated cells had distinct LC3 puncta, which represent LC3II conjugated to autophagosomal membranes (Figure 7C(Fig. 7)). Furthermore, western blotting confirmed that MMV652103 treatment led to the conversion of LC3 to LC3II (Figure 7D(Fig. 7)). Interestingly, there was an inhibition of total LC3 at the ½ IC_50_ concentration at both 24 and 48 h of treatment. We speculate that this result is due to MMV652103 exerting differential effects on autophagy with ½ IC_50_ inhibiting LC3-I, and consequently its conversion to LC3-II, and IC_50_ MMV652103 treatment enhancing autophagy. It is important to note that although the conversion of LC3 to LC3II is a good marker for the induction of autophagic structures it does not provide sufficient information about autophagic flux, which is the measure or rate of autophagic degradation. Considering this, additional experiments with autophagy inhibitors such as bafilomycin A1 and chloroquine, would need to be performed to confirm autophagic flux in response to MMV652103 treatment. Furthermore, when LC3 was depleted in MCF7 cells, MMV652103 was unable to induce cleaved PARP (Figure 7E(Fig. 7)). These results suggest that MMV652103-induced breast cancer cell death occurs through crosstalk between autophagy and apoptosis. 

### MMV652103 reduces MCF7 cell induced breast tumour weight and metastases in chick embryos

**Figure 8:**
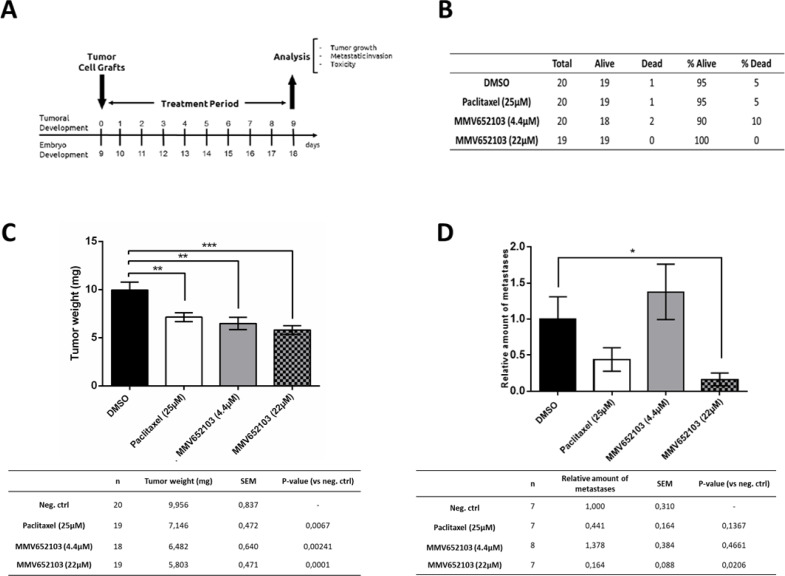
*In vivo* chick embryo studies of MMV652103. (A) Description of the chick embryo study. At day 10 (E10), tumors were detectable. Tumors were treated for a period of 10 days, every 48 h (E10, E12, E14, E16) by dropping 10 μl of vehicle (0.25% DMSO in PBS), 25 µM Paclitaxel (positive control), and MMV652103 at 4.4 and 22 µM. Subsequent analyses included assessment of tumour growth, metastatic invasion, and toxicity to the embryo. (B) Number of dead and surviving embryos for the different experimental groups at E18. (C) Mean values ± SEM of tumor weight measured for each experimental group. (D) The relative amount of metastases, measured by qPCR for Alu sequences in the lower CAM of groups, compared to negative control (arbitrary value for metastasis set to 1). *p <0.05, **p < 0.01, ***p < 0.001, student's t test.

We next investigated the cytotoxic activity of 4.4 μM and 22 μM MMV652103 in xenograft tumor experiments using chick embryos (Figure 8A(Fig. 8)). These concentrations were 2 and 10 times higher than the *in vitro* IC_50_ because unlike cells in culture, the chick embryo has an active metabolism and can catabolize and clear a compound. Concentrations higher than the IC_50 _obtained for a compound *in vitro* is therefore required to observe efficacy in the xenograft *in ovo*. Our results show that MMV652103 treatment did not result in an abnormal death ratio (Figure 8B(Fig. 8)), nor did it lead to any macroscopic abnormalities (results not shown). These data provide preliminary indication that MMV652103 treatment may be associated with minimal side effects. Importantly, the tumor weight was reduced by 34.89 % and 41.71 % in the 4.4 μM and 22 μM MMV652103 treated groups respectively which, while not significant, was more effective than that observed for 25 μM paclitaxel which resulted in only a 28.22 % reduction (Figure 8C(Fig. 8)). To determine the effect of MMV652103 on breast cancer cell metastases, genomic DNA was extracted from the lower chorioallantoic membrane (CAM) and analyzed by qPCR with specific primers for Alu sequences. The rationale for this was that the tumor cells were grafted onto the upper CAM and during metastasis the cancer cells invade the epithelium and basement membrane of the upper CAM and move through connective tissue into the vasculature into the lower CAM. Alu tandem repeats are only present in higher primates and therefore the quantification of human Alu sequences was used as a surrogate marker for metastasis in our xenograft model. The results show that 22 μM MMV652103 treatment resulted in a significant reduction in metastasis and was more effective than 25 μM paclitaxel (Figure 8D(Fig. 8)). 

## Discussion

Breast cancer is the most common malignancy in women worldwide and almost 50 % of breast cancer cases and 58 % of breast cancer related deaths occur in less developed countries. It remains a global health burden primarily due to late diagnosis, the development of tumor drug resistance, poor therapy response and debilitating side effects associated with current therapies (Massarweh and Schiff, 2006[53]; Cella and Fallowfield, 2008[14]; Redig and McAllister, 2013[69]). This highlights the critical need for more effective and affordable therapeutic interventions. This study adopts a drug repositioning strategy which refers to cases where drugs which are active in one disease is derivatized or used as a template for the synthesis of compounds active in another disease. Indeed, the anti-plasmodial diaryl-imidazopyridazine (MMV652103) compound was tested for its anti-breast cancer activity and several lines of evidence are provided to suggest that it may be an effective chemotherapeutic agent to treat breast cancer.

An effective anti-cancer therapeutic is proposed to have a sub-toxic treatment concentration and selectively targets cancer cells over normal somatic cells. In this study, the cytotoxicity of MMV652103 was tested in several breast cancer cell lines with different receptor status and results show that MMV652103 was most potent in the ER+ cell lines, MCF7 and T47D, where it exhibited an average IC_50_ of 2.5 µM. These results are encouraging because the anti-cancer drug screen criteria set by the National Cancer Institute (NCI) states that a potential therapeutic agent should have an initial single dose effect below 10 µM, after which, the ability of the drugs to inhibit additional cancer hallmarks can be assessed (Burger and Fiebig 2014[12]). Furthermore, MMV652103 was shown to be selective against MCF7 and T47D ER+ breast cancer cells with selectivity indices greater than 2 which indicates that it may be associated with fewer side-effects (Koch et al., 2005[39]; Badisa et al., 2009[5]). 

One of the major challenges with anti-breast cancer therapies is tumor recurrence and relapse which is in part due to tumor cells retaining their clonogenicity post-treatment (Fiebig et al., 2004[24]). It is therefore worth noting that the results from clonogenic cell survival assays show that MMV652103 significantly reduced the capacity of breast cancer cells to retain their reproductive ability or clonogenicity. The clonogenic assay is a valuable *in vitro* tool used in the drug discovery pipeline as it allows for an extended period of analysis and therefore serves as a reliable indicator of the effect of drugs on the reproductive integrity of cancer cells. Furthermore, literature has shown that data from clonogenic assays have significant correlations with clinical responses to drugs with correct predictions of 62 % for drug sensitivity and 92 % for drug resistance (Fiebig et al., 2004[24]). Furthermore, it is estimated that approximately 90 % of cancer-related deaths are due to cancer cell metastasis and the formation of secondary tumors (Bogenrieder and Herlyn, 2003[9]; Lazebnik, 2010[43]; Hanahan and Weinberg, 2011[32]; Guan, 2015[31]; Gandalovičová et al., 2017[26]). Due to the importance of this process, the impact of MMV652103 on cellular migration was measured and results showed that MMV652103 significantly inhibited the migratory ability of MCF-7 and T47D ER+ breast cancer cells. This suggests that MMV652103 has the potential to reduce or inhibit ER+ breast cancer cell metastases.

There is a growing appreciation for the need to understand the mechanisms of action of novel anti-cancer therapeutics. This will enable the stratification of patients and the identification of biomarkers to facilitate clinical research. Furthermore, it will support and optimize combination therapies and allow for the anticipation of potential resistance mechanisms (Cattley and Radinsky, 2004[13]). This study therefore explored the mechanisms of anti-cancer activity of MMV652103 and showed that it increased the production of ROS. Numerous studies have reported on the ROS-inducing effects of well-known chemotherapeutics in a variety of cancers. For example, Lee et al. (2000[46]) showed that the anti-estrogen tamoxifen, commonly used in the treatment of ER+ breast cancer, induced a slow and sustained increase in intracellular ROS levels and subsequent apoptosis in HepG2 human hepatoblastoma cells (Lee et al., 2000[46]). Another study showed that tamoxifen promoted the generation of ROS and the senescence phenotype in MCF7 breast cancer and HCT116 human colorectal carcinoma cells (Lee et al., 2014[45]). Moreover, the widely used chemotherapeutic, doxorubicin, has been shown to induce apoptosis via the generation of H_2_O_2_ in leukemia cells, while in osteosarcoma cells it was shown to increase intracellular H_2_O_2_ and superoxide levels (Tsang et al., 2003[79]; Mizutani et al., 2005[55]). It is reasonable to speculate that the ROS induced by MMV652103 is, in part, responsible for its ability to induce DNA DSBs and activate the canonical DNA damage repair pathway as well as the p38 MAPK pathway. Furthermore, these pathways may be upstream of MMV652103 induced apoptotic and autophagic cell death. Encouragingly, MMV652103 was able to activate both the extrinsic and intrinsic apoptotic pathways. Typically, it is the mitochondrial or intrinsic pathway of apoptosis that is the most deregulated form of cell death in cancer and a major contributor of chemoresistance. Therefore, activation of the extrinsic pathway is a desirable characteristic of these compounds, although this may be cell type-dependent (Lopez and Tait, 2015[50]; Pfeffer and Singh, 2018[65]). Tumours also often initially respond to pro-apoptotic therapies but gain the ability to bypass apoptosis which contributes to anti-cancer drug resistance and ultimately tumour recurrence (Wong, 2011[88]). Therefore, our results showing that in addition to apoptosis, MMV652103 was also possibly inducing autophagic cell death suggests that it would be more efficacious (Giansanti et al., 2011[29]).

Our results showing that MMV652103 inhibits the PI3K and PI4K family members, PIK3C2G and PI4KB respectively are interesting because these lipid kinases play an important role in cancer metabolism and have been identified as druggable targets especially to overcome acquired drug resistance (Engelman, 2009[23]; Chu et al., 2010[16]; Li et al., 2010[48], 2014[49]; Pinke and Lee, 2011[67]; Boller et al., 2012[10]; Juvekar et al., 2012[36]; Martini et al., 2013[52]; Ilboudo et al., 2014[34]; Waugh, 2014[86]; Andrs et al., 2015[2]). For example, PI4KB is co-amplified with PIPK1A, AKT3 and PIK3C2B (all localized to chromosome 1q) in over 60 % of human breast tumor samples where this quartet of phosphoinositide metabolizing genes was postulated to enhance phosphoinositide signaling resulting in increased cell proliferation and motility and a more aggressive phenotype (Waugh, 2014[86]). Indeed, PI4KB was found to drive the development of all breast cancer subtypes and was shown to co-operate with the small Rab GTPase, Rab11a, to promote breast cancer oncogenesis via a novel Akt activation pathway (Morrow et al., 2014[57]; Silva et al., 2015[75]). Furthermore, in triple negative breast cancer a high mutation frequency was identified in PI3K signaling pathway-related genes, including PIK3C2G, and HER2+ breast cancer cells were sensitized to trastuzamub treatment when PIK3C2G was silenced (Lapin et al., 2014[41]; Weisman et al., 2016[87]). Not surprisingly, the development of novel inhibitors of the PI3K and PI4K family of lipid kinases as targeted anti-cancer agents has gained traction (McPhail and Burke, 2020[54]). 

The *in vivo* anti-cancer potential of MMV652103 was assessed using the chick embryo chorioallantoic membrane (CAM) model. MMV652103 displayed a favouable safety profile since even after 10 days of treatment with ten times the *in vitro* IC_50_ there were no abnormal death ratios or macroscopic abnormalities observed in the chick embryos. These results suggest that MMV652103 may be associated with minimal side-effects. Promisingly, consistent with the *in vitro *data from this study, MMV652103 reduced MCF7 tumor weight as well as tumor cell metastases. Although the chick embryo model serves as a first *in vivo* proof-of-concept and is a powerful tool to eliminate early non-efficacious drug candidates, further analyses using alternative *in vivo* models such as the mouse model, would need to be employed to determine the pharmacokinetics, efficacy, and toxicity of MMV652103 more rigorously before proceeding to clinical trials.

## Conclusion

The data presented here show that MMV652103 may be a promising candidate for repositioning as an anti-breast cancer agent because it displays potent and selective cytotoxicity in MCF7 and T47D ER+ breast cancer cells, inhibited the migratory ability of these cells, induced apoptotic and autophagic cell death. Importantly, *in vivo *MMV652103 displayed a favorable safety profile and reduced MCF7 tumor weight and metastasis.

## Declaration

### Acknowledgments

This work was supported by grants from the National Research Foundation of South Africa (S.P.), the South African Research Chairs Initiative of the Department of Science and Innovation, administered through the South African National Research Foundation (K.C.), the Cancer Association of South Africa (S.P.), the Medical Research Council of South Africa (S.P. and K.C.) and the University of Cape Town (S.P. and K.C.). The content is solely the responsibility of the authors and does not necessarily represent the official views of the funding agencies.

### Conflict of interest

The authors declare that they have no known competing financial interests or personal relationships that could have appeared to influence the work reported in this paper.

## Figures and Tables

**Table 1:**
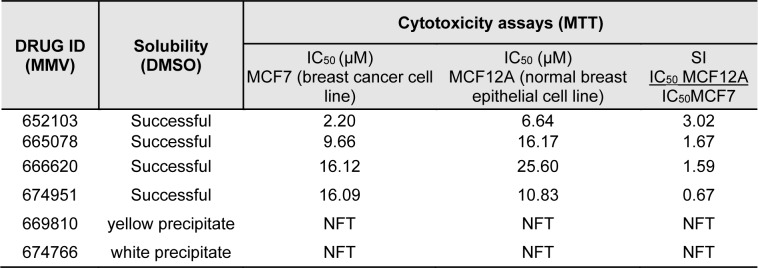
Solubility, cytotoxicity, and selectivity of MMV compounds in MCF7 breast cancer and non-malignant MCF12A breast epithelial cell lines were determined using the MTT cell viability assay. The data shown are from three independent experiments each performed in quadruplicate. NFT = no further testing, SI = Selectivity index
